# CD133-regulated nucleophosmin expression in human colon cancer cells

**DOI:** 10.1186/1878-5085-5-S1-A45

**Published:** 2014-02-11

**Authors:** Kun Kim, Kyung-Hee Kim, Seung Cheol Kim, Jun Pyu Hong, Won Ki Kim, Byong Chul Yoo, Ja-Lok Ku

**Affiliations:** 1 Colorectal Cancer Branch, Division of Translational and Clinical Research I, Research Institute, National Cancer Center, Gyeonggi, 410-769, Republic of Korea; 2 Laboratory of Cell Biology, Cancer Research Institute, Seoul National University College of Medicine, Seoul, 110-744, Republic of Korea; 3 Division of Gynecologic Oncology, Department of Obstetrics and Gynecology, Ewha Womans University Mokdong Hospital, Ewha Womans University School of Medicine, Seoul, 158-710, Republic of Korea

## 

Cancer stem cells (CSCs) are known to be resistant to conventional chemotherapy and radiotherapy. Specific CSC targeting and eradication is therefore a therapeutically important challenge. CD133 is a colorectal CSC marker with unknown function(s). Assessing proteomic changes induced by CD133 expression may provide clues not only to new CD133 functions but also to the chemotherapy and radiation susceptibility of colon cancer cells. To identify the proteins affected by CD133 expression, CD133-positive (CD133+) and CD133-negative (CD133–) human colon cancer cells were obtained by cell sorting. Whole proteomes were profiled from SW620/CD133+ and SW620/CD133– cells and analyzed by two-dimensional-based proteome analysis. Nucleophosmin (NPM1) was identified as a protein regulated by CD133. CD133 expression was not affected by NPM1, and an interaction between the two proteins was not observed. CD133 and NPM1 expression was positively correlated in 11 human colon cancer cell lines. However, the CD133+ subpopulation percentage or its value normalized against CD133 expression was only linked to intrinsic susceptibility of human colon cancer cells to 5-fluorouracil. The present study suggests that CD133-regulated NPM1 expression may provide a clue to novel CD133 function(s) linked to human colon cancer cell susceptibility to chemotherapy and radiation therapy.

